# A Capacitive DC-DC Boost Converter with Gate Bias Boosting and Dynamic Body Biasing for an RF Energy Harvesting System

**DOI:** 10.3390/s23010395

**Published:** 2022-12-30

**Authors:** Jiho Jung, Ickjin Kwon

**Affiliations:** Department of Electrical and Computer Engineering, Ajou University, Suwon 16499, Republic of Korea

**Keywords:** RF energy harvesting, DC-DC converter, boost converter, charge pump, high efficiency

## Abstract

In this paper, a fully integrated capacitive DC-DC boost converter for ultra-low-power internet of things (IoT) applications operating with RF energy harvesting is proposed. A DC-DC boost converter is needed to boost the low output voltage of the RF energy harvester to provide a high voltage to the load. However, a boost converter operating at a low voltage supplied by ambient RF energy harvesting has a problem in that power conversion efficiency is significantly lowered. The proposed on-chip capacitive DC-DC boost converter simultaneously applies gate bias boosting and dynamic body biasing techniques using only the internal boosted voltage without an additional circuit that increases power loss to boost the voltage, achieving high efficiency at an input voltage as low as 0.1 V. The designed capacitive boost converter achieves a peak power conversion efficiency (PCE) of 33.8% at a very low input voltage of 0.1 V, a 14% improvement over the peak PCE of the conventional cross-coupled charge pump. A maximum peak PCE of 80.1% is achieved at an input voltage of 200 mV and a load current of 3 μA. The proposed capacitive boost converter is implemented with a total flying capacitance of 60 pF, suitable for on-chip integration.

## 1. Introduction

Energy harvesting is one of the core technologies for battery-free, ultra-low-power internet of things (IoT) applications. RF energy harvesting technology can supply power to IoT devices by obtaining energy from ambient RF signals such as Wi-Fi signals, wireless cellular communication signals, and TV signals without a separate battery and power source [1,2,3]. In an RF energy harvesting system, an RF energy harvester obtains DC voltage from an RF signal received from an antenna. However, the output DC voltage that can be obtained from an RF energy harvester is limited because the power of the ambient RF signal is quite low. Therefore, there is a need for a power management circuit that includes a DC-DC boost converter to boost the voltage of the RF energy harvester to power the IoT device. The power management circuit is required to achieve high power conversion efficiency in low voltage input operations to provide power to the output by boosting the low DC voltage input from the RF-DC converter.

**Figure 1:**
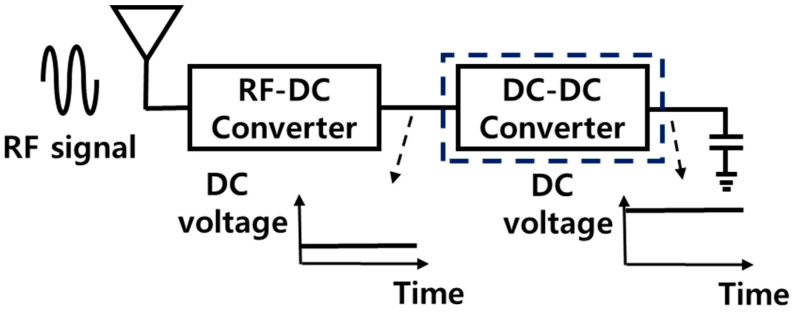
Block diagram of an RF energy harvesting system for ultra-low-power IoT applications.

Figure 1 shows a block diagram of an RF energy harvesting system for ultra-low-power IoT applications. In an RF energy harvesting system, an RF-DC converter is required to convert the RF signal of a wide frequency band into a DC signal. In addition, a DC-DC boost converter is required to maximize power conversion efficiency by lowering power loss in a low input voltage operation.

Capacitive DC-DC boost converters are suitable for on-chip integration because they do not require the off-chip inductors used in conventional inductor-based boost converters [4]. A DC-DC boost converter driven by the output voltage of an RF-DC converter for RF energy harvesting increases the conduction loss of the CMOS switch at an input voltage as low as 0.1 V, which significantly reduces the power conversion efficiency. Therefore, several methods have been proposed to improve the conductance of CMOS switches to improve the power conversion efficiency of the charge pump in low input voltage operations [5,6,7,8]. By increasing the clock amplitude using the clock booster circuit or by applying a fixed body bias to control the threshold voltage of the MOSFET due to the body effect, the conductance of the switch can be increased. However, booster circuits for clock boosting have limitations when operating at very low voltages, such as at 100 mV, and power conversion efficiency decreases due to additional power consumption. To reduce the on-resistance of the switch, a method of applying fixed forward body biasing voltage to an NMOS switch using the DC voltage inside the charge pump has been reported [5,8]. However, there is a disadvantage in that leakage current increases due to a decrease in the threshold voltage. A charge pump applying dynamic body biasing has been proposed [6], but it is difficult to operate at a low voltage of 0.1 V, and there is a limit to on-chip implementation because a large off-chip capacitor is used.

In this paper, a fully integrated on-chip capacitive DC-DC boost converter is proposed for IoT device applications operating with RF energy harvesting. It is designed to obtain high power conversion efficiency at a low voltage of 0.1 V based on gate bias boosting and dynamic body biasing techniques using only the internal boosted voltage without an additional circuit that increases power loss to boost the voltage.

## 2. Cross-Coupled Charge Pump

**Figure 2:**
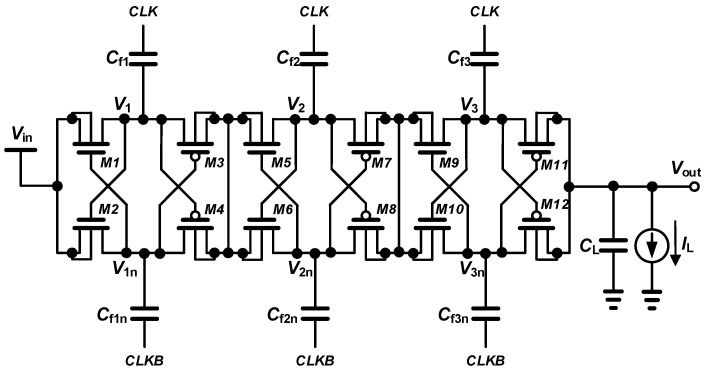
Schematic of a three-stage cross-coupled charge pump.

Figure 2 shows the circuit diagram of a three-stage cross-coupled charge pump (CCPP). *CLK* and *CLKB* have a phase difference of 180 degrees from each other and are applied to the gates of the switch transistors with the amplitude of the input voltage (*V*_in_) to control the switch to be on or off and charge or discharge the flying capacitor. In the cross-coupled charge pump circuit, the NMOS *M*_1_ and *M*_2_ turn on when the gate voltage reaches 2 *V*_in_ and turn off when the gate voltage reaches *V*_in_. On the other hand, when the gate voltage of PMOS *M*_3_ and *M*_4_ becomes *V*_in_, it turns on, and when it reaches 2 *V*_in_, it turns off. The body of the NMOS switch is connected to the source to avoid the body effect using deep n-well CMOS technology.

The output capacitor is charged by the current supplied through the upper path for half a cycle and is charged by the current supplied through the lower path for the other half cycle. Due to this, a shorter settling time and higher efficiency can be expected. The input current applied to the input during one cycle is expressed as

(1) $$I_{\mathrm{in}}=\left[\left(N+1\right)+\alpha \frac{f_{\mathrm{CLK}}\;C_{\mathrm{fly}}\;N}{I_{L}}V_{\mathrm{in}}\right]I_{L}$$

where *N* is the number of stages of the charge pump, *V*_in_ is the input voltage, *I*_L_ is the load current, *f*_CLK_ is the clock frequency, *C*_fly_ is the flying capacitance, and *α* is a constant representing the effect due to the parasitic component of the flying capacitor [8]. The steady-state output voltage of an *n*-stage charge pump is provided by

(2) $$V_{\mathrm{out}}=\left(N+1\right)\;V_{\mathrm{in}}-\frac{NI_{L}}{2f_{\mathrm{CLK}}\;C_{\mathrm{fly}}}$$

The voltage conversion efficiency (VCE) of a charge pump is defined as the ratio of the actual output voltage to the ideal output voltage. The power conversion efficiency (PCE) of a charge pump is defined as the ratio of the output power to the input power. The power losses in a charge pump are mainly caused by conduction losses, switching losses, and reverse leakage losses. Conduction losses increase proportionally to the channel resistance when the MOSFET switch is on. Increasing the width of the MOSFET reduces the on-channel resistance, which reduces conduction losses. On the other hand, the switching loss increases in proportion to the switching frequency and capacitance. Increasing the width of the MOSFET increases the capacitance, which increases the switching losses. There is a trade-off between conduction loss and switching loss as conduction loss decreases with switch width, while switching loss increases with switch width. Therefore, an optimal switch width value exists to minimize the total power dissipation [9].

**Figure 3:**
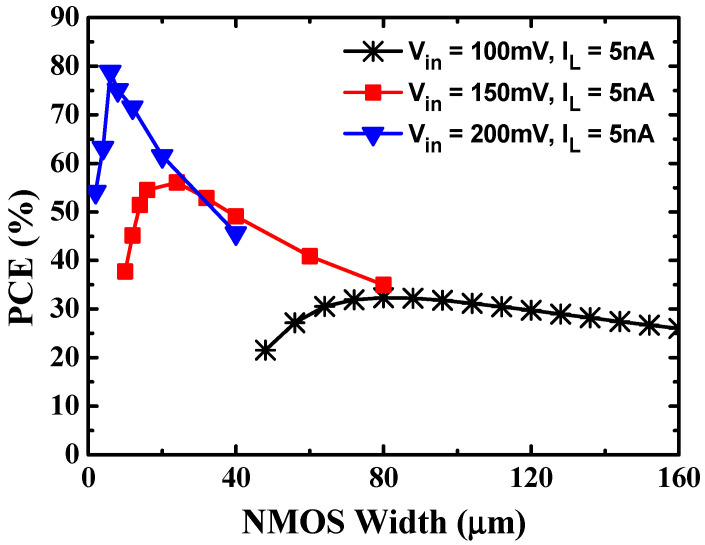
PCE according to NMOS switch width of a one-stage cross-coupled charge pump.

Figure 3 shows the PCE according to the NMOS switch width of a single-stage cross-coupled charge pump for different input voltages at the same load current of 5 nA. In the cross-coupled charge pump, the width of the PMOS switch is designed to be 2.5 times the width of the NMOS switch, which is the optimal value to obtain the maximum PCE. As shown in Figure 3, as the input voltage increases to 100 mV, 150 mV, and 200 mV, the optimal NMOS switch widths that maximize the PCE decrease to 80 μm, 24 μm, and 6 μm, respectively. The optimum MOSFET switch width to achieve maximum power conversion efficiency depends on the input voltage. In this work, to obtain high power conversion efficiency at a low input voltage, an optimal NMOS switch width is designed with a width of 80 μm to obtain the maximum PCE at an input voltage of 100 mV.

## 3. Proposed DC-DC Boost Converter Design

In a multi-stage charge pump, as the stage increases, the voltage level of the internal node of each stage increases step by step as much as the amplitude of the clock voltage. To increase the gate-source voltage of the MOS switch, the charge pump circuit is configured to apply the optimal gate bias level using the voltage of the internal node that increases at every stage. At low input voltage, performance degradation due to conduction loss is greater than performance degradation due to leakage, so the gate-source voltage of the MOS switch is increased to reduce conduction loss. In the proposed on-chip capacitive DC-DC boost converter, high power conversion efficiency at a low voltage of 0.1 V is achieved by applying gate bias boosting (GBB) and dynamic body biasing (DBB) techniques using the internal boosted voltage without an additional circuit that increases power loss to boost the voltage.

**Figure 4:**
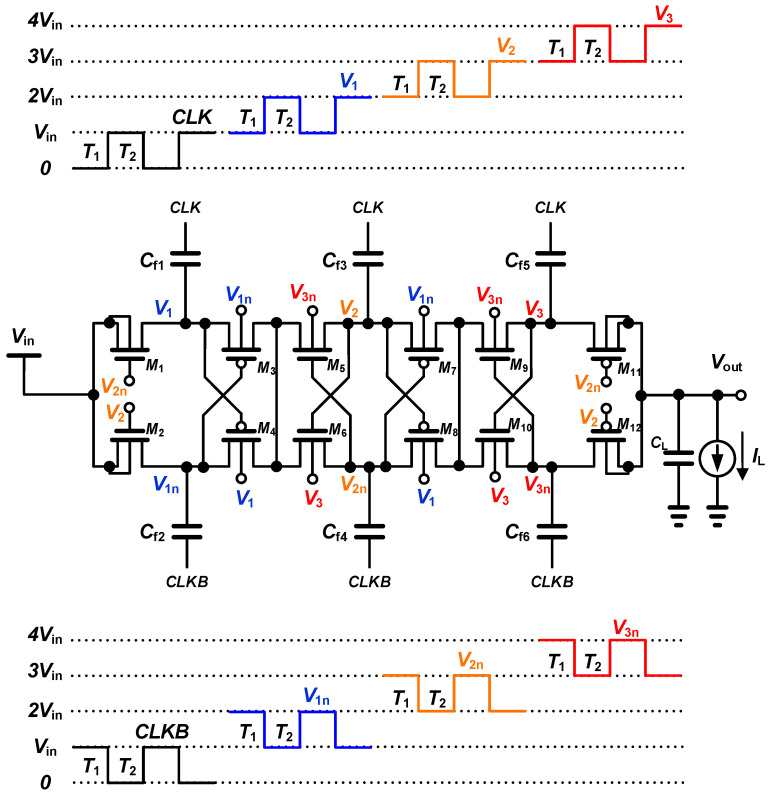
Schematic of the proposed three-stage charge pump using gate bias boosting and dynamic body biasing techniques.

Figure 4 shows the schematic of the proposed three-stage charge pump using GBB and DBB techniques with only the internal boosted voltage. In the proposed design, the GBB technique is applied to NMOS *M*_1_ and *M*_2_ in the first stage and PMOS *M*_11_ and *M*_12_ in the third stage, and the DBB technique is applied to other transistors. For the GBB, the gate terminals of NMOS switches *M*_1_ and *M*_2_ are connected to nodes *V*_2n_ and *V*_2_, respectively, and the gate terminals of PMOS switches *M*_11_ and *M*_12_ are connected to nodes *V*_2n_ and *V*_2_, respectively.

During the time interval of *T*_1_, the gate-source voltage of *M*_1_ becomes 2 *V*_in_, and the switch is turned on. On the other hand, during the time interval of *T*_2_, the gate-source voltage of *M*_2_ becomes 2 *V*_in_, and the switch is turned on. Since the turn-on, the gate-source voltage of the NMOS switch is 2 *V*_in_, which is doubled compared with conventional cross-coupled charge pumps, so the conduction losses of the switch are significantly reduced in a low-input voltage operation. As the gate voltage of the NMOS switch increases, conduction losses decrease, but leakage losses increase. When the gate terminals of *M*_1_ and *M*_2_ are connected to node *V*_2n_ and node *V*_2_, respectively, the total loss, including conduction and leakage losses, is minimized, and power conversion efficiency is maximized. In the same way as the NMOS switches, the turn-on gate-source voltage of the PMOS switches *M*_11_ and *M*_12_ is −2 *V*_in_, which is doubled compared with conventional cross-coupled charge pumps, so the conduction losses of the switch are significantly reduced in a low-input voltage operation. When the gate terminals of *M*_11_ and *M*_12_ are connected to nodes *V*_2n_ and *V*_2_, respectively, the total loss, including conduction and leakage losses, is minimized, and power conversion efficiency is maximized.

When the threshold voltage of a MOSFET used as a switch element is low, the switch-on resistance decreases so that the power conversion efficiency increases, and the settling time decreases in a low-voltage operation. However, in the switched-off state, the reverse leakage current increases due to the low threshold voltage. When the threshold voltage is high, the switch-on resistance increases, and the settling time increases, but the leakage current decreases in the switch-off state.

By using the body biasing of the MOSFET with deep n-well technology, the on-resistance and off-resistance of the switch can be adjusted by controlling its threshold voltage. The threshold voltage of NMOS, considering the effect of the body effect, is expressed as

(3) $$V_{\mathrm{TH}}=V_{\mathrm{TH}0}+\gamma \left(\sqrt{\left|2\phi_{F}\right|+V_{\mathrm{SB}}}-\sqrt{\left|2\phi_{F}\right|}\;\right)$$

where *V*_SB_ is the source-body voltage, *V*_TH0_ is the threshold voltage when *V*_SB_ = 0, *γ* is the body effect coefficient, and 2 *ϕ*_F_ is the strong inversion surface potential [9]. In the case of NMOS, the threshold voltage decreases in forward body biasing where the body voltage is higher than the source voltage. On the other hand, in PMOS, the threshold voltage decreases in forward body biasing where the body voltage is lower than the source voltage.

By applying different body bias voltages when the switch MOSFETs are in on or off states using the DBB technique, it is possible to lower the threshold voltage when it is on and increase the threshold voltage when it is off, thereby reducing conduction loss and leakage loss, respectively. Compared with the fixed body biasing method, the DBB technique alleviates the trade-off between conduction loss and leakage loss due to the threshold voltage.

In the proposed charge pump circuit, the threshold voltage of the switch MOSFET in on and off operations is controlled differently by applying the DBB technique. The charge pump circuit is configured to apply the optimal body bias level using the voltage of the internal node, which increases step by step as much as the amplitude of the input clock voltage. The DBB technique is applied to the remaining MOSFETs, except for *M*_1_, *M*_2_, *M*_11_, and *M*_12_, where the GBB technique is applied.

The body terminals of NMOS switches *M*_5_ and *M*_6_ are connected to nodes *V*_3n_ and *V*_3_, respectively. During the time interval of *T*_1_, the body voltage of *M*_5_ becomes 4 *V*_in_, and the body voltage of *M*_6_ becomes 3 *V*_in_. On the other hand, during the time interval of *T*_2_, the body voltage of *M*_5_ becomes 3 *V*_in_, and the body voltage of *M*_6_ becomes 4 *V*_in_. When NMOS switches *M*_5_ and *M*_6_ are turned on, the body-source voltage becomes 2 *V*_in_, and the threshold voltage is reduced. Therefore, conduction losses are reduced by significantly reducing the on-resistance of the switch in a low input voltage operation. On the other hand, when switches *M*_5_ and *M*_6_ are turned off, the body-source voltage becomes *V*_in_, and the threshold voltage is relatively higher than when the switches are turned on. Therefore, the off-resistance is increased, and the leakage current is reduced.

To apply the DBB technique in the same way, the body terminals of PMOS switches *M*_7_ and *M*_8_ are connected to nodes *V*_1n_ and *V*_1_, respectively. When switches *M*_7_ and *M*_8_ are turned on, the body-source voltage becomes −2 *V*_in_, and the threshold voltage is reduced. Therefore, conduction losses are reduced by significantly reducing the on-resistance of the switch in a low input voltage operation. On the other hand, when switches *M*_7_ and *M*_8_ are turned off, the body-source voltage becomes −*V*_in_, and the threshold voltage is relatively higher than when the switches are turned on, so the leakage current is reduced.

The body terminals of NMOS switches *M*_9_ and *M*_10_ are connected to nodes *V*_3n_ and *V*_3_, respectively. When *M*_9_ and *M*_10_ are turned on, the body-source voltage becomes *V*_in_ to reduce the threshold voltage, so conduction loss is reduced. The body terminals of PMOS switches *M*_3_ and *M*_4_ are connected to nodes *V*_1n_ and *V*_1_, respectively. When *M*_3_ and *M*_4_ are turned on, the body-source voltage becomes −*V*_in_ to reduce the threshold voltage, so conduction loss is reduced.

## 4. Simulation Results and Comparison

**Figure 5:**
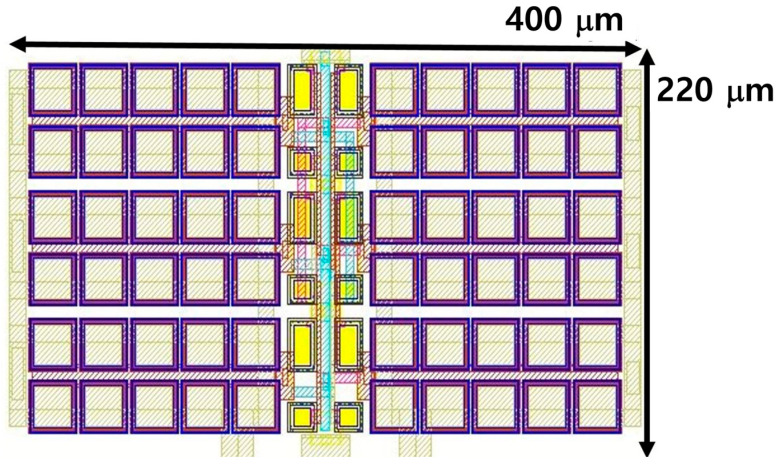
Layout of the proposed three-stage charge pump-based boost converter.

The proposed, fully integrated on-chip capacitive DC-DC boost converter based on a three-stage charge pump was designed using 0.18 μm CMOS technology. Figure 5 shows the layout of the proposed three-stage charge pump-based boost converter. The total silicon area is 0.088 mm^2^. To verify the effectiveness of the proposed charge pump and compare it with the conventional cross-coupled charge pump, post-layout simulations using Spectre were carried out.

**Figure 6:**
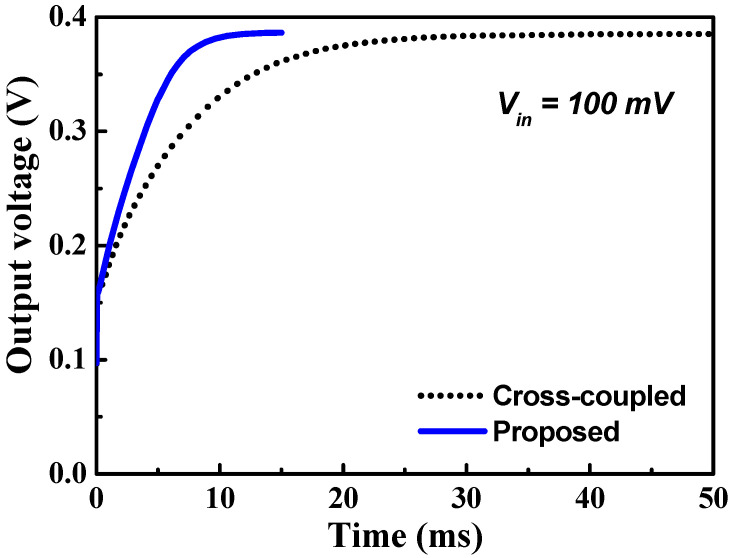
Simulated output voltage of the three-stage cross-coupled charge pump and the proposed charge pump.

Figure 6 shows the simulated output voltage of the three-stage cross-coupled charge pump and the proposed charge pump for an input voltage of 100 mV. The settling time of the proposed charge pump is 8.9 ms, which is a 57.8% reduction compared with the cross-coupled charge pump.

**Figure 7:**
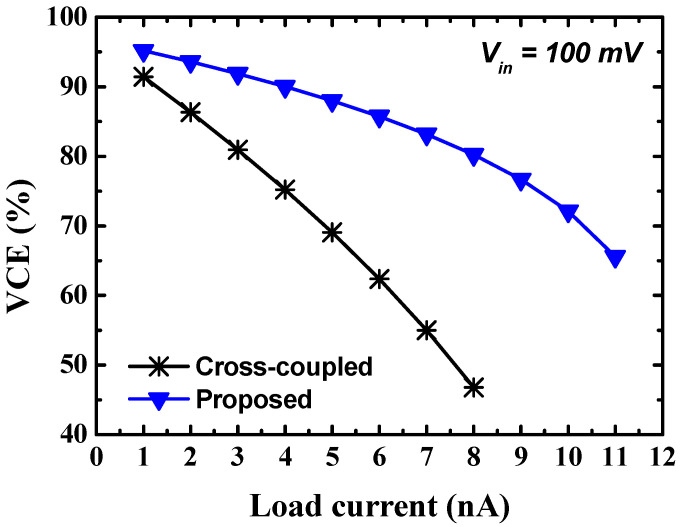
Simulated VCE according to the load current of the cross-coupled charge pump and proposed charge pump.

Figure 7 shows the simulated VCE according to the load current of the three-stage cross-coupled charge pump and the proposed charge pump for an input voltage of 100 mV. A clock is applied with a frequency of 1 MHz and a peak-to-peak amplitude of 100 mV. Each flying capacitance is 10 pF, and the total flying capacitance is 60 pF. As shown in Figure 6, as the load current increases, the output voltage decreases. When the GBB and DBB techniques are applied, the on-resistance of the switch is reduced, and the output voltage drop is reduced under the same load current condition, thereby improving the VCE under a wide load current condition.

**Figure 8:**
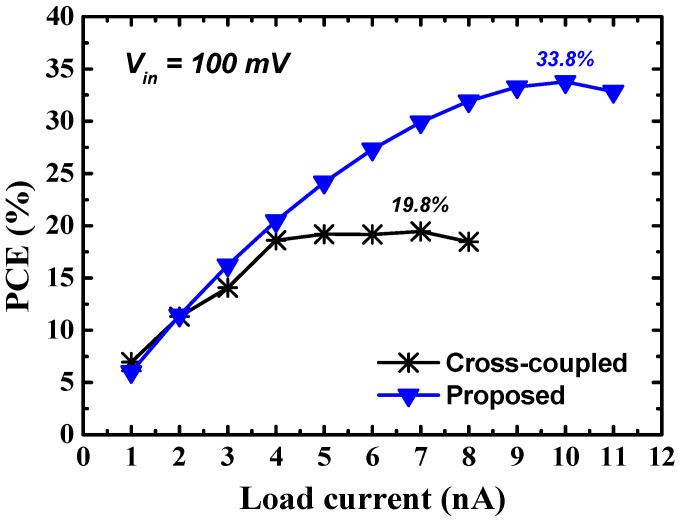
Simulated PCE according to the load current of the cross-coupled charge pump and the proposed charge pump.

Figure 8 shows the simulated PCE according to the load current of the three-stage cross-coupled charge pump and the proposed charge pump for an input voltage of 100 mV. The proposed three-stage charge pump—by applying both the GBB and DBB techniques using the internal boosted voltage—achieves a peak PCE of 33.8%, resulting in a significant PCE improvement of 14% compared with the cross-coupled charge pump.

**Figure 9:**
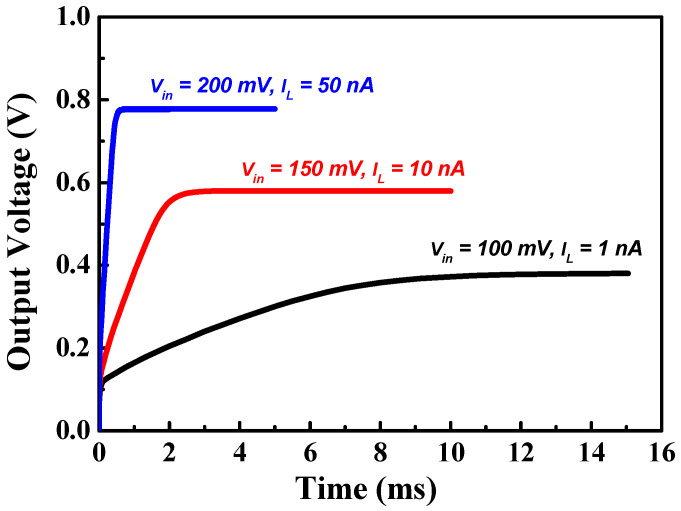
Transient output voltage of the proposed charge pump under various input voltage and load current conditions.

Figure 9 shows the transient output voltage of the proposed charge pump under various input voltage and load current conditions. The settling time is 8.9 ms for an input voltage of 100 mV and a load current of 1 nA. The settling time decreases as the input voltage increases, and the settling time decreases to 0.195 ms for an input voltage of 200 mV and a load current of 50 nA.

**Figure 10:**
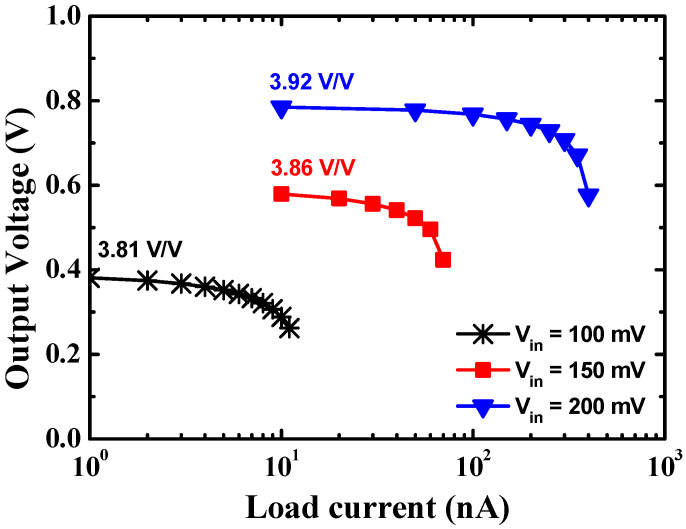
Output voltage according to the load current in different input voltages of the proposed charge pump.

**Figure 11:**
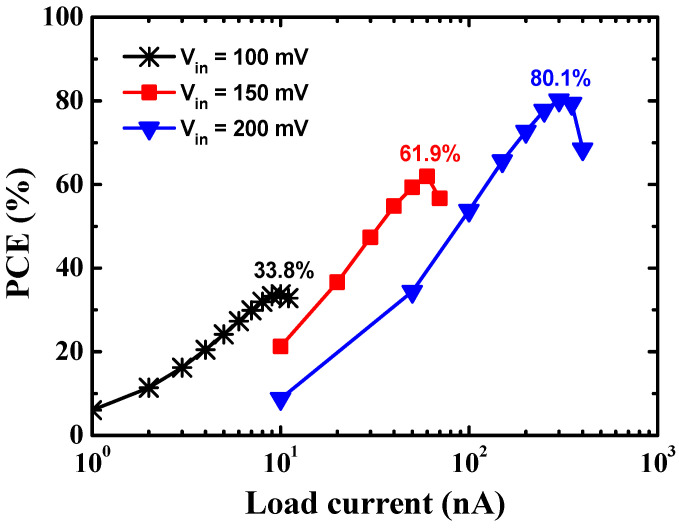
PCE according to the load current in different input voltage conditions of the proposed charge pump.

Figure 10 shows the output voltage according to the load current at the different input voltages of the proposed charge pump. The output voltage of the converter drops as the output load current increases, as expected by (2). Figure 11 shows the PCE according to the load current at different input voltages of the proposed charge pump. When the output voltage drops rapidly as the load current increases, the output power decreases, and the PCE of the converter drops. Thus, there is an optimum load current that achieves a peak PCE, and the PCE drops as the load current further increases. A peak PCE of 33.8% is achieved at an input voltage of 100 mV and a load current of 10 nA. A maximum peak PCE of 80.1% is achieved at an input voltage of 200 mV and a load current of 3 μA.

Table 1 summarizes the performance of the proposed capacitive boost converter and compares the performance with other works. The proposed on-chip capacitive DC-DC boost converter simultaneously applies the gate bias boosting (GBB) and dynamic body biasing (DBB) techniques using only the internal boosted voltage without an additional circuit that increases power loss to boost the voltage, achieving high efficiency at an input voltage as low as 0.1 V. The proposed charge pump based on the GBB and DBB techniques achieves a peak PCE of 35.3% at a very low input voltage of 0.1 V, which improves the peak PCE by 15.5% compared with the cross-coupled charge pump designed without applying the proposed circuit technique. In addition, it is implemented with a total flying capacitance of 60 pF, so it is suitable for on-chip integration and can be applied to IoT devices driven by RF energy harvesting.

Compared with other published works, a high PCE is achieved at the lowest input voltage of 0.1 V and implemented with a total flying capacitance of 60 pF, making it suitable for on-chip integration compared with [11]. The charge pump in [8] achieves the highest peak PCE but has a relatively high minimum input voltage of 150 mV. Since PCE can be improved by using advanced CMOS technology, which provides a lower threshold voltage, a higher PCE can be obtained by applying the proposed circuit technique using the advanced CMOS technology used in other works.

## 5. Conclusions

In this paper, a fully integrated on-chip capacitive DC-DC boost converter for IoT devices operating with RF energy harvesting is proposed. To achieve high power conversion efficiency at a low input voltage operation of 0.1 V, the proposed capacitive boost converter adopts the GBB and DBB techniques using only the internal boosted voltage without an additional circuit that increases power loss to boost the voltage. The proposed capacitive boost converter achieves a peak PCE of 33.8% at an input voltage of 0.1 V, which is 14% higher than the peak PCE of a conventional cross-coupled charge pump. A maximum peak PCE of 80.1% is achieved at an input voltage of 200 mV and a load current of 3 μA. The proposed capacitive boost converter is implemented with a total flying capacitor of 60 pF, enabling on-chip integration.

## Figures and Tables

**Table 1:** Performance summary and comparison.

Reference	This Work	[5]	[8]	[10]	[11]
CMOS process	180 nm	130 nm	28 nm FD-SOI	65 nm	65 nm
No. of stages	3	3	3	3	10
Technique	GBB and DBB	DBB	FBB	Bootstrap	Gate Boosting
Clock frequency	1 MHz	0.25 MHz	0.084 MHz	15.2 MHz	1 MHz
Total capacitor	60 pF	60,000 pF (off-chip)	31 pF	22.5 pF	1001 pF
Min. input voltage	100 mV	180 mV	150 mV	150 mV	100 mV
Peak PCE	33.8% @*V*_in_ = 100 mV	34% @*V*_in_ = 180 mV	34.9% @*V*_in_ = 150 mV	38.8% @*V*_in_ = 150 mV	33% @*V*_in_ = 100 mV

## Data Availability

Not applicable.
